# Long-term oral ACEI/ARB therapy is associated with disease severity in elderly COVID-19 omicron BA.2 patients with hypertension

**DOI:** 10.1186/s12879-023-08913-6

**Published:** 2023-12-18

**Authors:** Zhe Zhang, Shengyong Wu, Zhiyong Wang, Yue Wang, Hui Chen, Cheng Wu, Lize Xiong

**Affiliations:** 1 Department of Anesthesiology and Perioperative medicine, Shanghai Key Laboratory of Anesthesiology and Brain Functional Modulation, Clinical Research Center for Anesthesiology and Perioperative Medicine, Translational Research Institute of Brain and Brain-Like Intelligence, Shanghai Fourth People’s Hospital, School of Medicine, Tongji University, Shanghai, 200434 China; 2 Department of Military Health Statistics, Naval Medical University, Shanghai, 200433 China; 3 Department of Information Management, School of Medicine, Shanghai Fourth People’s Hospital, Tongji University, Shanghai, 200434 China

**Keywords:** Angiotensin-converting enzyme inhibitors, Angiotensin receptor antagonists, COVID-19, Hypertension, Aged patients

## Abstract

**Objective:**

To explore the effects of long-term oral ACEIs/ARBs on the incidence of exacerbation and in-hospital mortality in elderly COVID-19 Omicron BA.2 patients with hypertension, especially patients aged 80 years or older.

**Materials and methods:**

In this retrospective study, patients suffering mild and rcommon COVID-19 with hypertension who were hospitalized in the Shanghai Fourth People’s Hospital between April 2022 and June 2022 were enrolled. Primary outcomes included the incidence of exacerbation and in-hospital mortality. Secondary outcomes included the incidence of respiratory failure of patients, use of mechanical ventilation, nucleic acid conversion time (NCT), hospitalization costs, and the temporal trend of the incidence of exacerbations and in-hospital mortality in different age groups. The data were analysed using propensity score weighting (PSW).

**Results:**

In the entire cohort, there were 298 ACEI/ARB users and 465 non-ACEI/ARB users. The ACEI/ARB group showed a lower incidence of exacerbation (OR = 0.64, 95% CI for OR: 0.46–0.89, *P* = 0.0082) and lower in-hospital mortality (OR = 0.49, 95% CI for OR: 0.27–0.89, *P* = 0.0201) after PSW. Sensitivity analysis obtained the same results. The results of the subgroup of patients aged 80 years and older obtained a similar conclusion as the whole cohort. Most of the study indicators did not differ statistically significantly in the subgroup of patients aged 60 to 79 years except for rates of mechanical ventilation and respiratory failure.

**Conclusion:**

Antihypertensive therapy with ACEIs/ARBs might reduce the incidence of exacerbation and in-hospital mortality. The findings of this study support the use of ACEIs/ARBs in COVID-19 patients infected by Omicron BA.2, especially in patients aged 80 years or older with hypertension.

## Introduction

The coronavirus disease 2019 (COVID-19) pandemic is caused by the novel coronavirus SARS-CoV-2. Angiotensin-converting enzyme 2 (ACE2) is not only an enzyme but also a functional receptor on cell surfaces. SARS-CoV-2 enters host cells and causes ACE/ACE2 balance disruption and renin-angiotensin-aldosterone system (RAAS) activation. Some studies have shown that the expression of ACE2 and its regulation by conditions and potential complications may increase the susceptibility of tissues to COVID-19 [1]. Some researchers have proposed that the downregulation of ACE2 reduces susceptibility to SARS-CoV-2 infection in vitro, in vivo and human lungs and livers perfused ex situ by a series of model verification [2]. Angiotensin-converting enzyme inhibitors (ACEIs) and angiotensin II receptor blockers (ARBs) are RAAS inhibitors and have traditionally been used as first-line medications to treat hypertension [3, 4]. However, the use of ACEIs/ARBs in hypertensive patients with COVID-19 has aroused controversy. ACE2 is one of the issues at stake in the debate [5, 6]. Animal studies have demonstrated that ACEIs and ARBs can upregulate the expression of ACE2 [7]. Theoretically, such treatments could increase the risk of COVID-19 infection or exacerbate the severity of the disease. Several previous studies have shown that ACE2 expression is downregulated following SARS infection, which causes RAS overactivation and promotes pneumonia progression [8]. Oral treatment by ACEIs/ARBs could in turn inhibit the overactivation of RAS induced by the downregulation of ACE2 and thus prevent acute pulmonary injuries. Various cohort studies in different countries have investigated the relationship between ACEI or ARB treatment and severe outcomes of hospitalized COVID-19 patients, but the findings were inconsistent [9–14]. Richardson et al. [10] reported higher rates of mortality in patients on ACEIs/ARBs than in nonusers. In contrast, some evidence supports the benefit of using ACEIs or ARBs to potentially contribute to the improvement of clinical outcomes of COVID-19 patients with hypertension, and RAAS inhibitors might be associated with better COVID-19 prognosis [11, 12]. Some studies have proposed that ACEIs/ARBs are not associated with the severity and outcomes of COVID-19 infection in hospitalized patients with hypertension [13, 14].

The COVID-19 pandemic has even more drastic effects on elderly patients than on the general population. Large-scale clinical data have shown that elderly patients have a higher risk of COVID-19 incidence than the general population due to their advanced ages and medical comorbidities, such as hypertension and diabetes [15]. Most of the above studies have been conducted from the perspectives of mortality and infection rates, providing a reference basis for our clinical medication. The main focus of research so far has been on the early strain with stronger pathogenicity. In April 2022, the epidemic strain in Shanghai became Omicron, which has stronger transmission but weaker pathogenicity. We wanted to investigate whether COVID-19 Omicron is affected by ACEIs/ARBs.

During the COVID-19 pandemic, once severe cases occur, the lack of medical resources may lead to adverse outcomes for patients to consider. Preventing patients from transitioning from mild to severe is important. The purpose of this retrospective cohort study was to investigate the relationship between the use of ACEIs/ARBs and the occurrence of COVID-19 Omicron exacerbation and the in-hospital mortality of elderly patients with hypertension.

## Materials and methods

### Patients

This is a retrospective cohort study. All patients who were diagnosed with COVID-19 according to being tested positive using real-time reverse transcription polymerase chain reaction (RT‒PCR) testing via nasopharyngeal swabs were screened from April 12, 2022 to June 17, 2022 at Shanghai Fourth People`s Hospital, Shanghai, China. The cases in the study were classified as Omicron BA.2 by inference from previous studies which performed genomic analysis showing that there was a predominance of Omicron during the period evaluated [16, 17]. The inclusion criteria were age 60 years or older, diagnosed with essential hypertension, and taking antihypertensive medication regularly over 1 month before study inclusion. Patients admitted with asymptomatic COVID-19, with other reasons requiring oral ACEI, with survival time less than 48 h after admission, or time of diagnosis that could not be determined were excluded. Causing one of our primary outcomes was the incidence of exacerbations, and we excluded patients admitted with a diagnosis of severe or critical COVID-19. The Shanghai Fourth People’s Hospital Electronic Health Record was used to collect clinical information such as demographics, treatment, intraoperative data, pathology, and clinical outcomes.

The amended Helsinki Declaration commissioned this study. The study was approved by the Ethics Committee of the hospital (No. 2022-074-001) and reported in the Chinese Clinical Trial Register (No. ChiCTR2200061804). This study followed the Strengthening the Reporting of Observational Studies in Epidemiology (STROBE) reporting guidelines for cohort study.

### Exposure

We partitioned patients into ACEI/ARB and non-ACEI/ARB groups to investigate associations between ACEI or ARB use and outcomes in hypertensive populations. The ACEI/ARB group of oral medications containing ACEIs and ARBs. Patients receiving combination therapy which includes ACEIs/ARBs, belong to this group. The non-ACEI/ARB group of oral medications included CCBs and beta blockers but did not contain ACEIs/ARBs. The exposure of interest was prescription records indicating at least 1 month of prescription.

### Covariates

Baseline characteristics, demographic data, patient symptoms, medical history, and laboratory results of patients were collected, including patient age, sex, vaccination, symptoms, use of drugs, disease severity, history of immunological diseases, history of cerebrovascular disease, history of diabetes, history of coronary heart disease, history of chronic obstructive pulmonary disease, history of tumour, arrhythmias, heart failure, chronic kidney disease (CKD), oral hypoglycaemic agents (OAds), patient source, transfer to ICU or not, respiratory rate, body temperature, pulse, oxyhemoglobin saturation, blood pressure, use of aspirin, use of clopidogrel and other relevant covariates (Table 1).

**Table 1:** Patient Characteristics Before and After Propensity Score methods

Variables	Classification	Before PS methods						After PS Weighting						After PS Matching					
		All cohort (N = 763)	Non-ACEI group (N = 465)	ACEI group (N = 298)	*P* value	Statistic	SMD	All cohort (N = 1519)	Non-ACEI group (N = 760)	ACEI group (N = 759)	*P* value	Statistic	SMD	All cohort (N = 570)	Non-ACEI group (N = 285)	ACEI group (N = 285)	*P* value	Statistic	SMD
Sex	Male	309(40.50)	186(40.00)	123(41.28)	0.7263	0.12	0.02	627(41.28)	309(40.57)	319(42.00)	0.5718	0.32	0.03	239(41.93)	123(43.16)	116(40.70)	0.5524	0.35	0.01
	Female	454(59.50)	279(60.00)	175(58.72)				892(58.72)	452(59.43)	440(58.00)				331(58.07)	162(56.84)	169(59.30)			
Age, years		80.86 ± 9.84	81.46 ± 9.86	79.92 ± 9.73	0.0356	2.11	0.16	80.77 ± 13.88	80.80 ± 12.70	80.74 ± 15.55	0.9353	0.08	0.01	79.96 ± 9.79	79.86 ± 9.94	80.05 ± 9.67	0.8143	-0.24	0.03
Vaccination history, shots	0	522(68.41)	329(70.75)	193(64.77)	0.0957	7.89		1036(68.21)	519(68.24)	518(68.19)	0.9994	0.07		382(67.02)	193(67.72)	189(66.32)	0.9642	0.59	
	1	5(0.66)	2(0.43)	3(1.01)			0.07	10(0.68)	5(0.68)	5(0.67)			< 0.01	5(0.88)	2(0.70)	3(1.05)			< 0.01
	2	47(6.16)	24(5.16)	23(7.72)			0.10	96(6.31)	49(6.44)	47(6.19)			< 0.01	39(6.84)	19(6.67)	20(7.02)			0.01
	3	67(8.78)	33(7.10)	34(11.41)			0.15	139(9.13)	68(8.98)	70(9.27)			0.01	58(10.18)	27(9.47)	31(10.88)			0.01
	unknow	122(15.99)	77(16.56)	45(15.10)			0.04	238(15.67)	119(15.67)	119(15.68)			0.01	86(15.09)	44(15.44)	42(14.74)			0.01
Fever		173(22.67)	108(23.23)	65(21.81)	0.6491	0.21	0.03	338(22.25)	171(22.51)	167(21.99)	0.8049	0.06	0.02	126(22.11)	65(22.81)	61(21.40)	0.6864	0.16	0.06
Cough		460(60.29)	282(60.65)	178(59.73)	0.8013	0.06	0.01	918(60.41)	459(60.31)	459(60.50)	0.9405	0.01	0.02	345(60.53)	175(61.40)	170(59.65)	0.6683	0.18	0.01
Shortness of breath		37(4.85)	23(4.95)	14(4.70)	0.8762	0.02	0.01	80(5.25)	39(5.16)	40(5.33)	0.8794	0.02	0.01	28(4.91)	14(4.91)	14(4.91)	1.0000	0.00	0.03
Diarrhoea		10(1.31)	3(0.65)	7(2.35)	0.0435	4.08	0.14	21(1.36)	11(1.41)	10(1.30)	0.8444	0.04	0.01	5(0.88)	3(1.05)	2(0.70)	0.6533	0.20	0.06
Other symptoms		106(13.89)	56(12.04)	50(16.78)	0.0650	3.40	0.14	221(14.55)	111(14.60)	110(14.51)	0.9598	0.00	0.01	91(15.96)	44(15.44)	47(16.49)	0.7316	0.12	0.04
Use of Paxlovid		583(76.41)	365(78.49)	218(73.15)	0.0901	2.87	0.12	1169(76.92)	584(76.85)	584(76.98)	0.9515	0.00	< 0.01	419(73.51)	209(73.33)	210(73.68)	0.9244	0.01	0.01
Covid − 19 Classification	mild	366(47.97)	217(46.67)	149(50.00)	0.3690	-0.90	0.06	726(47.76)	360(47.37)	365(48.15)	0.8553	0.18	0.01	282(49.47)	141(49.47)	141(49.47)	1.0000	0.00	0.02
	common	397(52.03)	248(53.33)	149(50.00)				794(52.24)	400(52.63)	394(51.85)				288(50.53)	144(50.53)	144(50.53)			
Antoimmune disease		6(0.79)	5(1.08)	1(0.34)	0.2591	1.27	0.09	11(0.74)	6(0.79)	5(0.69)	0.8179	0.05	0.02	2(0.35)	1(0.35)	1(0.35)	1.0000	0.00	0.04
Diabetes		219(28.70)	130(27.96)	89(29.87)	0.5696	0.32	0.04	422(27.80)	212(27.89)	210(27.70)	0.9317	0.01	0.02	161(28.25)	79(27.72)	82(28.77)	0.7802	0.08	0.02
Coronary heart disease		230(30.14)	136(29.25)	94(31.54)	0.5001	0.45	0.04	454(29.86)	225(29.63)	228(30.10)	0.8431	0.04	0.02	171(30.00)	84(29.47)	87(30.53)	0.7839	0.08	0.01
Chronic obstructive pulmonary disease		16(2.10)	12(2.58)	4(1.34)	0.2441	1.36	0.09	35(2.30)	17(2.20)	18(2.40)	0.7938	0.07	0.01	9(1.58)	5(1.75)	4(1.40)	0.7369	0.11	0.08
Malignant disease		54(7.08)	37(7.96)	17(5.70)	0.2366	1.40	0.09	118(7.74)	55(7.17)	63(8.30)	0.4115	0.67	0.05	31(5.44)	15(5.26)	16(5.61)	0.8535	0.03	0.04
Patient Source	Community	548(71.82)	336(72.26)	212(71.14)	0.7379	0.11	0.02	1095(72.04)	551(72.43)	544(71.64)	0.7334	0.12	0.02	414(72.63)	209(73.33)	205(71.93)	0.7071	0.14	0.02
	Nursing home	215(28.18)	129(27.74)	86(28.86)				425(27.96)	210(27.57)	215(28.36)				156(27.37)	76(26.67)	80(28.07)			
Admission to ICU		36(4.72)	26(5.59)	10(3.36)	0.1553	2.02	0.11	70(4.61)	36(4.74)	34(4.48)	0.8076	0.06	0.01	21(3.68)	11(3.86)	10(3.51)	0.8240	0.05	< 0.01
Respiratory rate, per min		18.33 ± 1.77	18.30 ± 1.77	18.39 ± 1.77	0.4665	-0.73	0.06	18.30 ± 2.44	18.31 ± 0.21	18.3 ± 2.78	0.9307	0.09	< 0.01	18.41 ± 1.62	18.42 ± 1.42	18.40 ± 1.80	0.8367	0.21	0.03
Body temperature, °C		36.64 ± 0.40	36.65 ± 0.40	36.63 ± 0.40	0.3378	0.96	0.07	36.64 ± 0.55	36.64 ± 0.50	36.63 ± 0.63	0.8428	0.20	0.01	36.64 ± 0.39	36.64 ± 0.39	36.64 ± 0.39	0.9370	0.08	0.01
Heart rate, beats per min		76.35 ± 13.60	76.47 ± 13.90	76.16 ± 13.13	0.7597	0.31	0.02	76.04 ± 19.08	76.15 ± 17.63	75.92 ± 21.16	0.8159	0.23	0.01	76.13 ± 13.18	76.09 ± 13.18	76.17 ± 13.20	0.9393	-0.08	0.01
Pulse oxygen saturation, %		98.48 ± 1.36	98.44 ± 1.38	98.55 ± 1.34	0.2702	-1.10	0.08	98.44 ± 2.11	98.47 ± 1.68	98.41 ± 2.65	0.5369	0.62	0.07	98.51 ± 1.27	98.48 ± 1.20	98.54 ± 1.35	0.5546	-0.59	< 0.01
Diastolic blood pressure, mm Hg		73.69 ± 12.60	72.94 ± 12.88	74.86 ± 12.09	0.0396	-2.06	0.16	73.62 ± 17.74	73.68 ± 16.37	73.57 ± 19.71	0.9060	0.12	0.02	74.78 ± 12.41	74.87 ± 12.72	74.69 ± 12.11	0.8582	0.18	0.01
Systolic blood pressure, mm Hg		134.34 ± 21.60	134.06 ± 21.77	134.80 ± 21.36	0.6449	-0.46	0.04	134.15 ± 30.89	134.33 ± 27.74	133.98 ± 35.30	0.8280	0.22	0.02	134.88 ± 21.33	135.08 ± 21.41	134.68 ± 21.28	0.8231	0.22	0.03
WBC ×10^9^/L		5.67 ± 2.50	5.75 ± 2.39	5.53 ± 2.66	0.2312	1.20	0.09	5.67 ± 3.69	5.65 ± 2.91	5.69 ± 4.66	0.8258	-0.22	0.04	5.53 ± 2.38	5.50 ± 2.03	5.56 ± 2.70	0.7948	-0.26	< 0.01
PLT×10^9^/L		179.83 ± 66.13	178.88 ± 67.82	181.32 ± 63.50	0.6187	-0.50	0.04	177.90 ± 91.94	178.89 ± 85.40	176.90 ± 101.43	0.6725	0.42	0.03	179.74 ± 65.61	177.96 ± 67.71	181.53 ± 63.50	0.5171	-0.65	0.02
LYM%		25.86 ± 11.66	25.59 ± 11.52	26.29 ± 11.88	0.4201	-0.81	0.06	25.75 ± 16.58	25.86 ± 14.58	25.64 ± 19.32	0.7925	0.26	0.03	26.33 ± 11.53	26.39 ± 11.06	26.27 ± 1.99	0.8943	0.13	0.02
NEU%		63.70 ± 13.00	64.19 ± 12.97	62.94 ± 13.04	0.1965	1.29	0.10	63.82 ± 18.64	63.67 ± 16.31	63.98 ± 21.81	0.7480	-0.32	0.04	62.95 ± 12.71	62.85 ± 12.30	63.04 ± 13.13	0.8571	-0.18	0.02
LYM×10^9^/L		1.27(0.87–1.71)	1.28(0.90–1.71)	1.26(0.83–1.70)	0.5746	-0.56	0.07	1.26(0.85–1.67)	1.29(0.91–1.68)	1.26(0.80–1.67)	0.0589	1.89	0.01	1.28(0.87–1.70)	1.29(0.92–1.71)	1.26(0.84–1.70)	0.3524	0.93	0.02
NEU×10^9^/L		3.27(2.31–4.60)	3.36(2.34–4.68)	3.14(2.26–4.37)	0.0610	-1.87	0.10	3.21(2.29–4.56)	3.31(2.32–4.61)	3.18(2.26–4.55)	0.2020	1.28	0.03	3.13(2.28–4.43)	3.18(2.29–4.41)	3.07(2.27–4.44)	0.7457	0.32	0.02
PT(S)		11.40 ± 1.50	11.44 ± 1.63	11.33 ± 1.27	0.3253	0.98	0.08	11.43 ± 2.10	11.41 ± 2.01	11.45 ± 2.23	0.7452	-0.33	0.04	11.38 ± 1.38	11.41 ± 1.48	11.35 ± 1.29	0.5780	0.56	0.04
TT(S)		14.68 ± 3.18	14.75 ± 3.82	14.58 ± 1.77	0.4747	0.72	0.06	14.64 ± 3.90	14.67 ± 4.39	14.61 ± 2.97	0.7670	0.30	0.02	14.52 ± 2.10	14.47 ± 2.38	14.58 ± 1.77	0.5521	-0.59	0.06
APTT(S)		30.02 ± 3.46	30.03 ± 3.58	30.01 ± 3.28	0.9363	0.08	0.01	30.09 ± 4.90	30.02 ± 4.46	30.16 ± 5.52	0.5934	-0.53	0.03	30.05 ± 3.36	30.13 ± 3.41	29.97 ± 3.30	0.5722	0.57	0.01
D-dimer(mg/L)		0.66(0.43–1.08)	0.66(0.45–1.23)	0.66(0.41–0.90)	0.0090	-2.61	0.14	0.66(0.42–1.07)	0.66(0.42–1.13)	0.66(0.43–1.02)	0.4821	0.70	0.03	0.66(0.42–0.96)	0.66(0.43–1.04)	0.66(0.41–0.93)	0.2118	1.25	0.01
CRP (mg/L)		9.23(3.48–23.53)	9.41(3.87–24.96)	8.57(3.04–22.45)	0.1805	-1.34	0.06	9.07(3.43–23.70)	9.53(3.74–23.76)	8.65(3.09–23.68)	0.3237	0.99	0.05	8.57(3.38–21.99)	8.90(3.74–21.99)	8.33(3.04–21.86)	0.5262	0.63	0.03
IL-6 (pg/ml)		31.75(17.21–80.15)	31.75(17.24–93.90)	31.75(17.19–60.79)	0.1436	-1.46	0.06	31.75(17.19–81.38)	31.75(16.80-92.41)	31.75(17.32–72.43)	0.5355	0.62	0.16	31.75(15.21–78.30)	31.75(15.04–93.90)	31.75(17.19–63.07)	0.5111	0.66	0.02
PCT (ng/ml)		0.02(0.02–0.06)	0.02(0.02–0.07)	0.02(0.02–0.05)	0.4444	-0.76	0.04	0.02(0.02–0.06)	0.02(0.02–0.06)	0.02(0.02–0.07)	0.4974	-0.68	0.03	0.02(0.02–0.06)	0.02(0.02–0.07)	0.02(0.02–0.05)	0.6068	0.51	0.04
ALB(g/L)		38.87 ± 4.15	38.71 ± 4.29	39.11 ± 3.91	0.1936	-1.30	0.10	38.88 ± 5.81	38.90 ± 5.46	38.86 ± 6.32	0.8778	0.15	0.01	39.07 ± 4.04	38.98 ± 4.20	39.16 ± 3.89	0.5918	-0.54	0.04
ALT (U/L)		16.19(11.96–23.97)	16.19(12.35–24.06)	16.19(11.50-23.84)	0.6612	-0.44	0.03	16.19(11.88–24.36)	16.19(12.36–23.97)	16.19(11.57–25.42)	0.7009	-0.38	0.02	16.19(11.88–24.08)	16.19(12.48–24.30)	16.19(11.50-23.67)	0.5221	0.64	0.01
AST (U/L)		23.93(19.59–31.13)	23.93(19.92–32.34)	23.67(19.26–29.48)	0.0303	-2.17	0.11	23.93(19.67–31.57)	23.93(19.92–31.50)	23.93(19.59–31.98)	0.3436	0.95	0.02	23.93(19.51–30.32)	23.93(19.73–31.50)	23.61(19.16–29.41)	0.0876	1.71	0.02
TB (µmol/L)		10.82(8.39–14.89)	10.82(8.44–15.12)	10.82(8.39–14.60)	0.9020	-0.12	0.08	10.82(8.39–15.10)	10.82(8.24–15.12)	10.82(8.58–15.10)	0.2724	-1.10	0.03	10.82(8.37–14.75)	10.82(8.20–14.70)	10.82(8.60-14.75)	0.4085	-0.83	< 0.01
Cre(umol/L)		62.80(51.80–80.30)	62.80(51.60–76.00)	62.80(52.70–83.00)	0.0759	1.78	< 0.01	62.80(52.00-80.80)	62.80(51.20–75.20)	62.80(52.70–83.30)	0.0074	-2.68	0.03	62.80(51.60–80.50)	62.80(50.80–75.20)	62.80(52.70–81.80)	0.0758	-1.78	0.10
BNP (pg/mL)		74.00(60.00–74.00)	74.00(62.00–81.00)	74.00(54.00–74.00)	0.2124	-1.25	0.09	74.00(57.00–74.00)	74.00(60.00–74.00)	74.00(54.00–74.00)	0.9197	-0.10	< 0.01	74.00(55.00–74.00)	74.00(61.00–74.00)	74.00(53.00–74.00)	0.8352	0.21	< 0.01
Ctnl (ng/mL)		0.02(0.02–0.02)	0.02(0.02–0.03)	0.02(0.02–0.02)	0.3804	-0.88	0.02	0.02(0.02–0.02)	0.02(0.02–0.02)	0.02(0.02–0.03)	0.6409	-0.47	0.07	0.02(0.02–0.02)	0.02(0.02–0.02)	0.02(0.02–0.02)	0.5702	-0.57	0.02
CK-MB (ng/mL)		2.11(1.76–2.68)	2.11(1.75–3.05)	2.11(1.76–2.36)	0.0872	-1.71	0.13	2.11(1.75–2.61)	2.11(1.75–2.93)	2.11(1.74–2.43)	0.1455	1.46	0.02	2.11(1.72–2.48)	2.11(1.72–2.59)	2.11(1.74–2.36)	0.5049	0.67	0.04
GLU (mmol/l)		6.11 ± 2.50	6.00 ± 2.20	6.28 ± 2.92	0.1417	-1.47	0.11	6.12 ± 3.53	6.10 ± 3.00	6.13 ± 4.23	0.8858	-0.14	0.01	6.24 ± 2.64	6.25 ± 2.44	6.24 ± 2.83	0.9772	0.03	< 0.01
Hb (g/dl)		123.45 ± 18.29	123.49 ± 18.51	123.38 ± 17.97	0.9389	0.08	0.01	123.41 ± 25.94	123.40 ± 23.48	123.41 ± 29.41	0.9928	-0.01	0.01	123.23 ± 18.12	122.97 ± 18.13	123.49 ± 18.13	0.7326	-0.34	0.05
Cerebrovascular disease		169(22.15)	105(22.58)	64(21.48)	0.7201	0.13	0.03	334(21.93)	165(21.69)	169(22.17)	0.8200	0.05	0.01	125(22.16)	64(22.70)	61(21.63)	0.7610	0.09	0.03
Arrythmias		7(0.92)	6(1.29)	1(0.34)	0.1772	1.82	0.11	14(0.92)	7(0.92)	7(0.92)	0.9989	0.00	< 0.01	3(0.53)	2(0.71)	1(0.35)	0.5627	0.34	0.04
Heart failure		9(1.18)	7(1.51)	2(0.67)	0.2977	1.08	0.08	15(0.99)	9(1.16)	6(0.82)	0.5105	0.43	0.03	3(0.53)	1(0.35)	2(0.71)	0.5627	0.34	0.03
CKD		5(0.66)	4(0.86)	1(0.34)	0.3809	0.77	0.07	9(0.59)	5(0.65)	4(0.52)	0.7434	0.11	0.02	2(0.35)	1(0.35)	1(0.35)	1.0000	0.00	< 0.01
Use of OADs		151(19.79)	87(18.71)	64(21.48)	0.3493	0.88	0.07	290(19.04)	147(19.27)	143(18.81)	0.8177	0.05	0.01	116(20.57)	58(20.57)	58(20.57)	1.0000	0.00	< 0.01
Use of aspirin		124(16.25)	78(16.77)	46(15.44)	0.6250	0.24	0.03	258(16.97)	127(16.64)	131(17.30)	0.7308	0.12	0.02	97(17.20)	51(18.09)	46(16.31)	0.5769	0.31	0.05
Use of clopidogrel		124(16.25)	75(16.13)	49(16.44)	0.9087	0.01	0.01	255(16.75)	126(16.57)	129(16.93)	0.8508	0.04	0.01	91(16.13)	43(15.25)	48(17.02)	0.5671	0.33	0.05

Cause of the weight of observation after PS weighting may not be integer, the count of each group may less or more than the total of all group after rounding off.

### Outcomes

The incidence of exacerbations and in-hospital mortality in the two groups served as the primary outcomes. Assessment of disease status followed the guidelines of SARS-CoV-2 (The Ninth Trial Version of the Chinese National Health Commission): mild type, with slight clinical symptoms but no imaging presentation of pneumonia; common type, with fever, respiratory tract, and other symptoms, imaging findings of pneumonia; severe type, with any of the following conditions: respiratory distress, respiratory frequency ≥ 30 times/minutes, finger oxygen saturation at rest ≤ 93%, or oxygenation index [PaO2/FiO2] ≤ 300 mmHg (1 mmHg = 0.133 kPa), the clinical symptoms worsened progressively, and lung imaging showed that the lesions progressed significantly > 50% within 24 ~ 48 h; critical type, with any of the following conditions: respiratory failure requiring mechanical ventilation, shock, combined with other organ failure that requires intensive care unit care and treatment. Patients were considered to have an exacerbation if their disease status changed from mild or common to severe or critical during hospitalization. The incidence of exacerbations was defined as the proportion of patients who had an exacerbation among the total patients in the group. In-hospital mortality was defined as all-cause mortality during hospitalization, jointly and separately from COVID-19 infection.

The secondary outcomes included (1) the incidence and difference of respiratory failure of patients, use of mechanical ventilation, nucleic acid conversion time (NCT), and hospitalization costs in these two groups and (2) the temporal trend of the incidence of exacerbations and in-hospital mortality in different age groups.

### Statistical analysis

Continuous variables were expressed as means and standard deviations or median and interquartile ranges according to data distributions, and categorical variables were expressed as counts and percentages. Continuous, categorical, and ordinal variables were analysed with Student’s t-test, Pearson chi-square test or wilcoxon rank sum test, respectively. The inverse probability of treatment weighting (IPTW) method was used as Model 1 for propensity score weighting (PSW), which was used to account for the aforementioned confounders. The propensity score was determined using a logistic regression model with all covariates. Adequacy matching for no significant imbalance of each baseline covariate was assessed by standardized mean differences (SMDs), and |SMD| less than or equal to 0.1 means that there was no significant difference between the two groups. In subsequent analysis, unbalanced covariates were balanced by multivariable linear regression or logistic regression models.

We performed sensitivity analyses using propensity score matching (PSM) (Model 2) and multivariable regression analysis (Model 3) to evaluate the robustness of our findings. PSM was performed using the “greedy nearest-neighbour” algorithm and calculated the predicted probability of the ACEI/ARB group versus the non-ACEI/ARB group among all patients with 1:1 matching with a calliper distance of 0.2 of the standard deviation of the logit of the propensity score (Model 2). The aforementioned confounders for which the differences between the two groups were statistically significant were used to build the multivariable linear regression or logistic regression models as Model 3.

Subgroup analyses for all outcomes were carried out in the PSW cohort with patients aged 80 years and older and those aged 60 to 79 years. The Cochran-Armitage trend test was used to evaluate the significance of trends in the incidence of exacerbations and in-hospital mortality in different age groups.

All tests were two-tailed, and P < 0.05 was considered significant unless otherwise specified. Statistical analysis was performed using SAS version 9.4 (SAS Institute Inc.) and R version 4.0.4 (R Foundation for Statistical Computing).

## Results

**Fig. 1:**
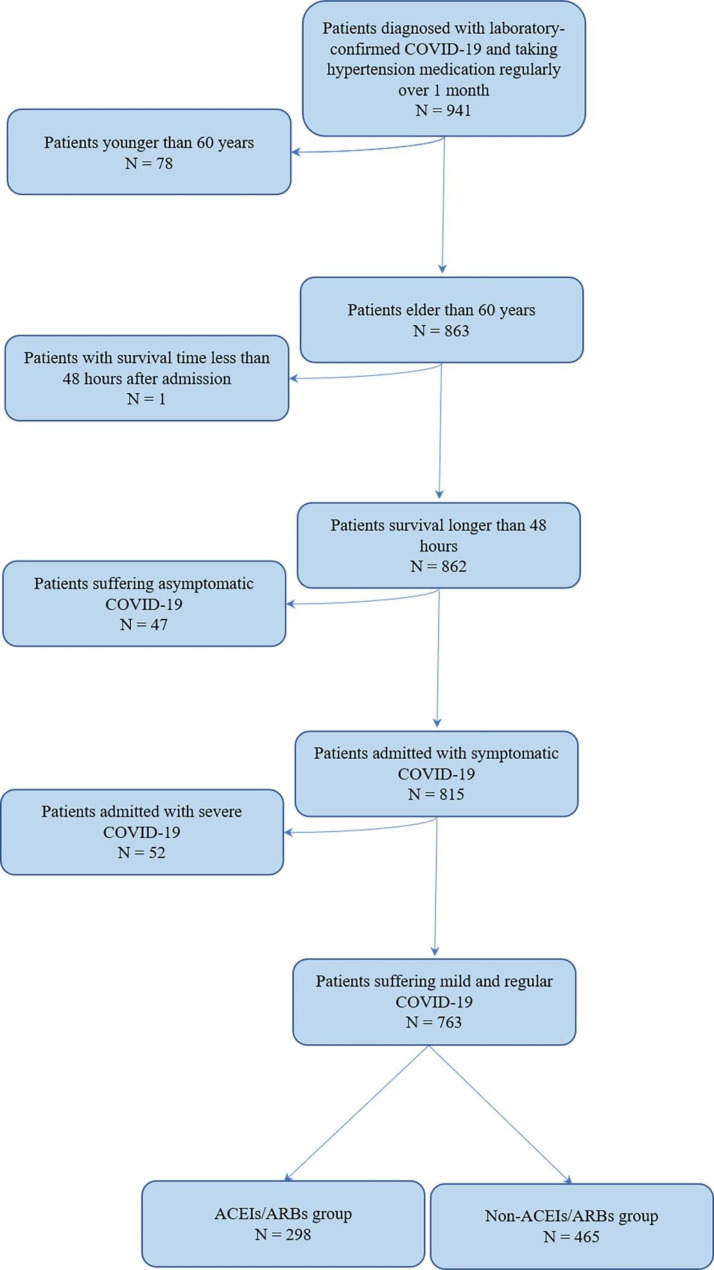
Selection flow diagram of target population

From April 2022 to July 2022, 941 patients diagnosed with laboratory-confirmed COVID-19 and taking hypertension medication regularly over 1 month were admitted to the Shanghai Fourth People`s Hospital. 78 patients were younger than 60, 1 patient had a survival time less than 48 h after admission, 47 patients were admitted with asymptomatic COVID-19, and 52 patients were admitted with severe and critical COVID-19 and were excluded according to the study protocol. Overall, 298 patients using ACEIs/ARBs and 465 patients using other drugs were included in the analysis (Fig. 1).

### Baseline characteristics

**Fig. 2:**
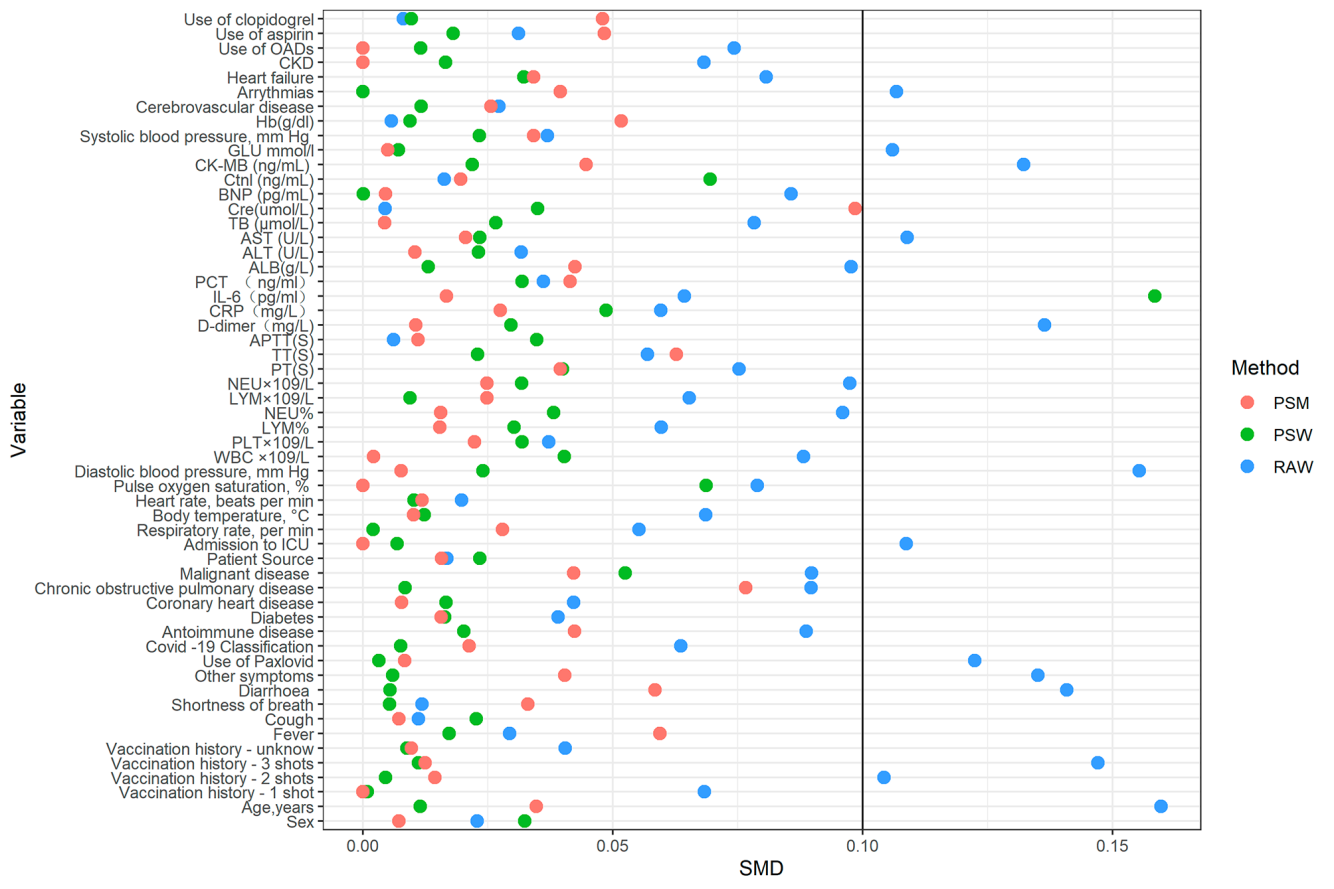
SMD of patients, patients after propensity score weighting, and patients after propensity score matching

Table 1 depicts the baseline characteristics of the entire cohort as well as the two groups of individuals (Table 1). Participants in the PSW and PSM included both those who used ACEIs/ARBs (n = 298) and those who did not (n = 465). There were 759 patients in the ACEI/ARB group and 760 patients in the non-ACEI/ARB group after PSW. Nearly all covariates were balanced between the two groups (SMD < 0.10), except the level of IL-6 (Table 1; Fig. 2).

### Primary outcomes

**Fig. 3:**
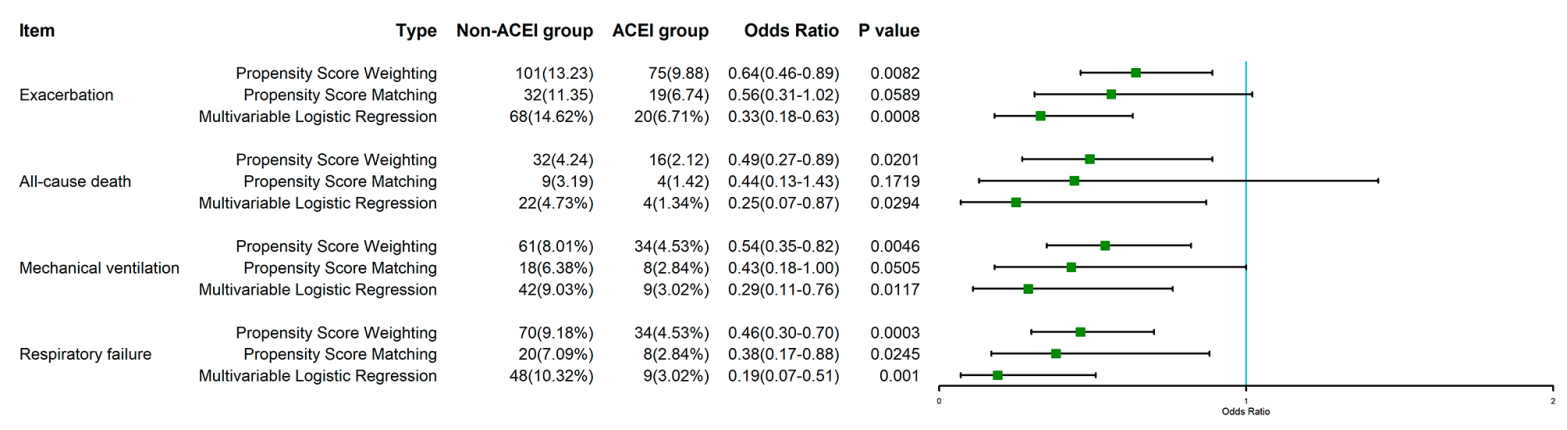
The of odd ratios (95% Confidence Interval) of outcomes

Overall, the crude incidence of exacerbation in the ACEI/ARB group was 6.71% (20/298), and that in the non-ACEI/ARB group was 14.62% (68/465). There was a significantly lower incidence of exacerbation in patients using ACEIs/ARBs during hospitalization compared with patients without the use of ACEIs/ARBs (9.88% (75/760) vs. 13.23% (101/763), OR = 0.64, 95% CI for OR: 0.46–0.89, *P* = 0.0082, Table 2; Fig. 3) after PSW.

**Table 2:** Relationship between drugs and outcomes of patients’

Variables	Propensity Score Weighting (Model 1)				Propensity Score Matching (Model 2)				Multivariable Regression (Model 3)	
	Non-ACEI group (N = 763)	ACEI group (N = 760)	OR/β(95%CI)	*P* value	Non-ACEI group (N = 282)	ACEI group (N = 282)	OR/β(95%CI)	*P* value	OR/β(95%CI)	*P* value
Exacerbation	101(13.23)	75(9.88)	0.64(0.46–0.89) ^a^	0.0082	32(11.35)	19(6.74)	0.56(0.31–1.02)^a^	0.0589	0.33(0.18–0.63) ^a^	0.0008
All-cause death	32(4.24)	16(2.12)	0.49(0.27–0.89) ^a^	0.0201	9(3.19)	4(1.42)	0.44(0.13–1.43)^a^	0.1719	0.25(0.07–0.87) ^a^	0.0294
Mechanical ventilation	61(8.01)	34(4.53)	0.54(0.35–0.82) ^a^	0.0046	18(6.38)	8(2.84)	0.43(0.18-1.00)^a^	0.0505	0.29(0.11–0.76) ^a^	0.0117
Respiratory failure	70(9.18)	34(4.53)	0.46(0.30–0.70) ^a^	0.0003	20(7.09)	8(2.84)	0.38(0.17–0.88)^a^	0.0245	0.19(0.07–0.51) ^a^	0.0010
Nucleic acid conversion time (NCT), days	14.00(10.00–19.00)	13.00(9.00–17.00)	-1.23(-2.21 - -0.26) ^b^	0.0132	14.00(10.00–19.00)	13.00(9.00–17.00)	-1.33(-2.44 - -0.21)^b^	0.0199	-0.91(-1.86-0.04) ^b^	0.0619
Total hospitalization costs, dollars	1458.21(991.93-2376.11)	1335.90(912.84-2109.78)	-124.16(-476.19–227.86) ^b^	0.4889	1333.01(968.67-2268.39)	1254.68(873.69-1886.94)	-204.07(-554.91–146.76)^b^	0.2537	-169.66(-412.67-73.34) ^b^	0.1716

^a^ Odds ratios; ^b^ β value

In the PSM cohort, the difference in exacerbation rate between the non-ACEI/ARB group and the ACEI/ARB group was not significant (6.74% (19/282) vs. 11.35% (32/282), OR = 0.56, 95% CI for OR: 0.31–1.02, *P* = 0.0589, Table 2; Fig. 3). The results of the multivariable logistic regression analysis were the same as those of Model 1 (OR = 0.33, 95% CI for OR: 0.18–0.63, *P* = 0.0008, Table 2; Fig. 3).

The in-hospital mortality of the ACEI/ARB group was 1.34% (4/298), and that of the non-ACEI/ARB group was 4.73% (22/465). In the primary analysis, patients who used ACEIs/ARBs had significantly lower in-hospital mortality than those who did not (2.12% (16/760) vs. 4.24% (32/763), OR = 0.49, 95% CI for OR: 0.27–0.89, *P* = 0.0201, Table 2; Fig. 3) after PSW. In Model 2, the difference in in-hospital mortality between the non-ACEI/ARB group and ACEI/ARB group was not statistically significant (1.42% (4/282) vs. 3.19% (9/282), OR = 0.44, 95% CI for OR: 0.13–1.43, *P* = 0.1719, Table 2; Fig. 3). Model 3 (multivariable logistic regression analysis) produced the same results as Model 1 (OR = 0.25, 95% CI for OR: 0.07–0.87, *P* = 0.0294, Table 2; Fig. 3).

### Secondary outcomes

The incidence of mechanical ventilation in the ACEI/ARB group was 3.02% (9/298), and that in the non-ACEI/ARB group was 9.03% (42/465). Patients who used ACEIs/ARBs had a significantly lower incidence of mechanical ventilation than those who did not (4.53% (34/760) vs. 8.01% (61/763), OR = 0.54, 95% CI for OR: 0.35–0.82, *P* = 0.0046, Table 2; Fig. 3) after PSW. In Model 2, the difference in in-hospital mortality between the non-ACEI/ARB group and ACEI/ARB group was not statistically significant (2.84% (8/282) vs. 6.38% (18/282), OR = 0.43, 95% CI for OR: 0.18-1.00, *P* = 0.0505, Table 2; Fig. 3). Model 3 (multivariable logistic regression analysis) produced the same results as Model 1 (OR = 0.29, 95% CI for OR: 0.11–0.76, *P* = 0.0117, Table 2; Fig. 3).

The incidence of respiratory failure in the ACEI/ARB group was 3.02% (9/298), and that in the non-ACEI/ARB group was 10.32% (48/465). Patients who used ACEIs/ARBs had a significantly lower incidence of respiratory failure than those who did not (4.53% (34/760) vs. 9.18% (70/763), OR = 0.46, 95% CI for OR: 0.30–0.70, *P* = 0.0003, Table 2; Fig. 3) after PSW. Model 2 (2.84% (8/282) vs. 7.09% (20/282), OR = 0.38, 95% CI for OR: 0.17–0.88, *P* = 0.0245, Table 2; Fig. 3) and Model 3 (OR = 0.19, 95% CI for OR: 0.07–0.51, *P* = 0.0010, Table 2; Fig. 3) came to the same result as Model 1.

The median COVID-19 NCT of all cohorts was 14 days (interquartile range = 10–18 days). In the PSW cohort, the median NCT of the ACEI/ARB group was significantly shorter than that of the non-ACEI/ARB group (13.0 (9.0–17.0) vs. 14.0 (10.0–19.0), β=-1.23, 95% CI for β: -2.21 - -0.26, *P* = 0.0132, Table 2), and the same conclusion was reached in the PSM cohort (13.0 (9.0–17.0) vs. 14.0 (10.0–19.0), β=-1.33, 95% CI for β: -2.44 - -0.21, *P* = 0.0199, Table 2). However, the difference between the 2 groups in multivariable linear regression analysis (β=-0.91, 95% CI for β: -1.86-0.04, *P* = 0.0619, Table 2) was not statistically significant.

The difference in total hospitalization costs between the ACEI/ARB group and the non-ACEI/ARB group (1335.90 (912.84-2109.78) vs. 1458.21 (991.93-2376.11), β=-124.16, 95% CI for β: -476.19–227.86, *P* = 0.4889, Table 2) was not significant in the PSW cohort.

**Fig. 4:**
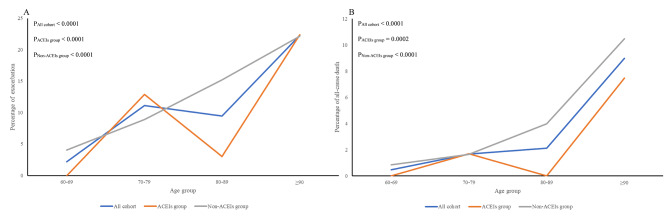
Age-specific incidence of exacerbation and all-cause death of elderly COVID-19 Omicron BA.2 patients with hypertension

The incidence of exacerbation and all-cause death increased from 60 to 69 years old to ≥ 90 years old (P_exacerbation_ < 0.0001, P_all−cause death_ < 0.0001, Table 3; Fig. 4) in the PSW cohort, and the trend test of both groups came to the same result (Table 3; Fig. 4).

**Table 3:** Age-specific trend test of the exacerbation and all-cause death incidence of elderly COVID-19 Omicron BA.2 patients with hypertension

Variables	Group	Statistic	*P* value
Exacerbation			
	All cohort	-6.5005	< 0.0001
	ACEIs group	-4.2134	< 0.0001
	Non-ACEIs group	-4.9226	< 0.0001
All-cause death			
	All cohort	-5.6431	< 0.0001
	ACEIs group	-3.6706	0.0002
	Non-ACEIs group	-4.2786	< 0.0001

### Subgroup analyses

In patients aged 80 years and older, the results of subgroup analyses were reasonably close to those of the primary analysis. The ACEI/ARB group had a significantly lower rate of exacerbation (10.64% (43/407) vs. 17.75% (78/441), OR = 0.56, 95% CI for OR: 0.37–0.83, *P* = 0.0039, Table 4), in-hospital mortality (2.93% (12/407) vs. 6.36% (28/441), OR = 0.45, 95% CI for OR: 0.23–0.90, *P* = 0.0235, Table 4), and incidence of respiratory failure (7.62% (31/407) vs. 12.42% (55/441), OR = 0.59, 95% CI for OR: 0.37–0.94, *P* = 0.0252, Table 4).

**Table 4:** Relationship between drugs and outcomes of patients’ in subgroup

Variables	Patients older than 80 years					Patients younger than 79 years				
	All cohort (N = 848)	Non-ACEI group (N = 441)	ACEI group (N = 407)	OR/β(95%CI)	*P* value	All cohort (N = 675)	Non-ACEI group (N = 322)	ACEI group (N = 353)	OR/β(95%CI)	*P* value
Exacerbation	122(14.33)	78(17.75)	43(10.64)	0.56(0.37–0.83) ^a^	0.0039	55(8.08)	23(7.06)	32(9.01)	0.90(0.48–1.68) ^a^	0.7454
All-cause death	40(4.71)	28(6.36)	12(2.93)	0.45(0.23–0.90) ^a^	0.0235	8(1.26)	4(1.34)	4(1.18)	0.89(0.23–3.45) ^a^	0.8634
Mechanical ventilation	77(9.06)	46(10.39)	31(7.62)	0.72(0.45–1.16) ^a^	0.1767	19(2.78)	15(4.76)	3(0.97)	0.16(0.05–0.57) ^a^	0.0046
Respiratory failure	86(10.11)	55(12.42)	31(7.62)	0.59(0.37–0.94) ^a^	0.0252	19(2.78)	15(4.76)	3(0.97)	0.16(0.05–0.57) ^a^	0.0046
Nucleic acid conversion time (NCT) days	15.00(11.00–19.00)	16.00(11.00–20.00)	14.00(10.00–18.00)	-1.34(-2.68-0.01) ^b^	0.0513	12.00(9.00–16.00)	12.00(9.00–18.00)	12.00(9.00–16.00)	-0.93(-2.3-0.45) ^b^	0.1861
Total hospitalization costs, dollars	1595.64(1073.72-2673.14)	1695.23(1075.64-2836.08)	1546.37(1072.97-2514.90)	34.50(-514.59-583.59) ^b^	0.9018	1175.18(859.81-1753.43)	1207.48(889.06-1837.47)	1142.40(851.43-1664.66)	-269.05(-639.62-101.51) ^b^	0.1541

^a^ Odds ratios; ^b^ β value

In the subgroup of patients aged 60 to 79 years, the ACEI/ARB group showed a lower incidence of mechanical ventilation (0.97% (3/353) vs. 4.76% (15/322), OR = 0.16, 95% CI for OR: 0.05–0.57, *P* = 0.0046, Table 4) and respiratory failure (0.97% (3/353) vs. 4.76% (15/322), OR = 0.16, 95% CI for OR: 0.05–0.57, *P* = 0.0046, Table 4) than the non-ACEI/ARB group.

## Discussion

In this retrospective study, our results suggest that ACEI or ARB treatment could reduce the incidence of exacerbation and possibly decrease the mortality of elderly COVID-19 patients with preexisting hypertension. After subgroup analysis, the main benefit was for hypertensive patients over 80 years old. In addition, the incidence of mechanical ventilation and respiratory failure in the ACEI/ARB group was significantly lower, and the median COVID-19 NCT in the ACEI/ARB group was shorter than that in the non-ACEI/ARB group. However, there was no statistically significant difference in the average hospitalization expenses between the two groups of patients. This finding supported the continued use of RAS inhibitors in COVID-19 Omicron patients with hypertension, which provides clinical evidence for the recommendations.

The effects of ACEIs/ARBs on clinical outcomes appeared to be influenced by RAAS, as the coronavirus SARS-CoV-2 entered the cells through ACE2 [18]. Previous animal studies have shown that ACEI/ARB treatment could upregulate the expression of the ACE2 receptor [19]. ACEIs/ARBs could elevate the level of ACE2 to exacerbate SARS-Cov2 infection. As a result, prolonged ACEI/ARB therapy may worsen patients’ COVID-19 duration. However, some fundamental studies and pathophysiological studies reported opposite findings [20, 21]. Long-term use of ARBs could block the detrimental effects of angiotensin II to prevent acute pulmonary injuries, such as pulmonary vasoconstriction, pulmonary vascular permeability elevation, inflammation, and interstitial fibrosis [21]. The severity of COVID-19 is associated with interleukin-6 (IL-6), C-reactive protein (CRP), and other proinflammatory factors [22, 23]. The binding of SARS-CoV-2 to host ACE2 results in the release of proinflammatory factors, which may harm vital organs through an IL-6-induced cytokine storm [24]. Previous studies have demonstrated that early intervention to alleviate such cytokine storms could improve the clinical outcomes of severe COVID-19 [25]. Patients with hypertension who take ACEIs or ARBs may produce less angiotensin II, express more ACE2, and have significantly reduced inflammatory cytokine production [25]. Meng et al. [13] observed that patients receiving ACEI or ARB therapy had a lower rate of severe diseases and a trend towards a lower level of IL-6 in peripheral blood. We made a speculative connection between the results and the use of ACEIs/ARBs to reduce cytokine storms, which then reduced the impairment of lung function and accelerated the speed of rehabilitation. A reduction in mortality might also be associated with this. This result is consistent with a study from a retrospective study in France that concluded that in very old subjects hospitalized in geriatric settings for COVID-19, mortality was significantly lower in subjects treated with ARB or ACEI before the onset of infection [26].

The severity of COVID-19 is associated with various risk factors, such as older age, male sex, and comorbidities [5, 6, 8, 15, 27]. Hypertension, coronary heart disease, diabetes, and advanced age could also increase susceptibility to SARS-CoV-2 infection [28, 29]. According to reports, COVID-19 patients generally have a mortality rate of 1–5%, but further analysis by age reveals that the mortality rate for patients aged 80 years and older may be as high as 14.8% [27]. A WHO report showed that after adjusting for the confounding effects of age, sex, ethnicity, prior infection, vaccination status, comorbidities, effect of province and effect of public/private sector, the Omicron variant had a reduced severity and lower mortality compared with the Delta variant [30]. Substitutions in the receptor-binding domain of Omicron may be associated with the enhanced affinity of S-protein to the ACE2 receptor, which might lead to the increased transmissibility of the Omicron variant [31]. Previous studies have mostly reported the impact of ACEI/ARB drugs on early strains, but we are more concerned about whether Omicron is also affected, especially in elderly individuals. The findings of this study showed that the mortality rate for patients aged 80 years and older was 4.71%. It was slightly lower in the ACEI/ARB group, at 2.93%. However, patients older than 80 years in the aged subgroup had a higher incidence of exacerbation and mortality than patients younger than 79 years, indicating that SARS-CoV-2 infection could lead to more severe outcomes in elderly patients, especially in patients aged 80 years and older. The results suggested that elderly patients with hypertension might choose antihypertensive drugs more carefully after COVID-19 infection.

ACEIs and ARBs are both RAAS inhibitors, but it is insufficient that our study does not differentiate between ACEIs and ARBs. Lumping them together might mask specific effects of one or the other. Some scholars have proposed that ARBs might be superior to ACEs for the treatment of hypertensive COVID-19 patients [32]. Research has shown that among hypertensive patients hospitalized for COVID-19, ARBs were associated with a lower crude rate of in-hospital mortality [33]. The differences between ACEIs and ARBs require further research.

Overall, these findings suggested potential beneficial effects observed with continued use of ACEI/ARB therapy in elderly COVID-19 Omicron BA.2 patients with hypertension. Other studies should also investigate whether ACEIs/ARBs were continued during hospitalization. Findings should be confirmed using other populations and study designs, including randomized controlled trials in both the general and younger populations.

### Limitations

As a retrospective study, there were several limitations in this study. First, due to the study’s retrospective design, not all confounding factors could be eliminated. BMI could be a key covariate, but the data were incomplete. Second, the study is a single-centre retrospective study. This design inherently has limitations in terms of generalizability. Third, due to insufficient data, patients could not be stratified based on when they used ACEIs/ARBs. Although various models could be used to adjust the confounding variables, other variables that could explain the severity of COVID-19 were possibly overlooked in this study. Finally, this study period is short, and we cannot be sure that the findings are not influenced by seasonal or other temporal factors. Although the findings of this study demonstrated that chronic exposure to ACEIs/ARBs was associated with better outcomes, the influences of these limitations should be considered.

## Conclusion

This study provided support for the continued use of ACEIs or ARBs by clinicians because they might slow the progression of mild and regular COVID-19 to severe COVID-19. The use of ACEIs/ARBs could benefit patients aged 60 years or older, especially patients aged 80 years or older.

## Data Availability

The datasets used or analysed during the current study are available from the corresponding author on reasonable request.
